# Cell-based high-throughput screens for the discovery of chemotherapeutic agents

**DOI:** 10.18632/oncotarget.513

**Published:** 2012-05-29

**Authors:** Jennifer T. Fox, Kyungjae Myung

**Affiliations:** ^1^ Genome Instability Section, Genetics and Molecular Biology Branch, National Human Genome Research Institute, National Institutes of Health, Bethesda, MD

**Keywords:** ATAD5-luciferase, genotoxins, DNA repair mutants, cancer, drug discovery

## Abstract

With modern advances in robotics and data processing, high-throughput screening (HTS) is playing an increasingly growing role in the drug discovery process. The ultimate success of HTS depends upon the development of assays that are robust and reproducible in miniaturized formats, have low false-positive rates, and can identify drugs that offer improvements over those currently on the market. One example of such an assay is the ATAD5-luciferase HTS assay, which identified three antioxidants that could kill cancer cells without inducing mutagenesis. Here we discuss the ATAD5-luciferase assay and expand upon the value of HTS in identifying other potential cancer drugs, focusing on cell-based assays that involve DNA damage or repair pathways.

## INTRODUCTION

High-throughput screening (HTS) is an automated process that allows for the rapid testing of large chemical, genetic, or biological libraries. It utilizes 96-, 384-, or 1536-well microplates, robotics, liquid handling devices, sensitive detectors, and data-processing software to identify a small number of effectors of a particular biological mechanism from collections that can contain up to two million drug candidates or leads. HTS has increasingly been used by both academic institutions and pharmaceutical companies to identify diagnostic biomarkers [1], expedite and reduce the costs associated with the discovery of new drugs, and screen FDA-approved compounds for additional uses, purposes, and indications.

HTS requires the development of robust assays with high signal-to-noise ratios that are adaptable to small volumes. These assays can be either biochemical (cell-free) or cell-based. Examples of biochemical assays include fluorescence resonance energy transfer (FRET), fluorescence correlation spectroscopy (FCS), fluorescence intensity distribution analysis (FIDA), and *in vitro* transcription assays [2]. Examples of cell-based assays include RNAi [3], second messenger, cell proliferation, and reporter assays [4]. Cell-based assays have several advantages over biochemical assays: they enable screens to be conducted in a context that more closely resembles a natural physiological state; in general, they are less costly and time-consuming in that they do not require purification of an active target protein; and they can immediately select against compounds that cannot permeate cellular membranes to reach intracellular targets, thereby eliminating additional validation steps. However, because they contain more than one target, cell-based assays may also require more complex secondary screens than HTS assays that use cell-free systems.

In this manuscript, we discuss the various cell-based HTS assays that have been developed to aid in the discovery of chemotherapeutic agents, focusing on those that exploit the DNA damage response. Such assays include tests for genotoxic agents, assays that utilize specific DNA repair mutants, and screens for compounds that can overcome chemoresistance.

### Screening for Genotoxic Compounds

Many chemotherapeutic treatments involve the administration of genotoxic compounds that damage DNA to the point of inducing cancer cell death via well-established DNA damage response signaling networks. Such genotoxins include alkylating agents (chlorambucil, cyclophosphamide), platinum drugs (cisplatin, oxalaplatin), antimetabolites (5-fluorouracil, methotrexate), anthracyclines (doxorubicin, daunorubicin), and topoisomerase inhibitors (topotecan, etoposide), all of which stall DNA replication, collapse replication forks, and produce DNA double-strand breaks (DSBs), resulting in the apoptosis of rapidly dividing cells [5]. There are currently several cell-based HTS assays available to identify genotoxic compounds, including GreenScreen HC GADD45a-GFP (Gentronix Ltd.), BlueScreen HC (Gentronix Ltd.), CellCiphr p53 (Cellumen Inc.), and CellSensor p53-bla (Invitrogen Corp.) [6-8]. However, cell-based genotoxicity assays are notorious for high false-negative rates due to lack of metabolic activation and the removal of genotoxic lesions by the DNA repair system, and the results generated from the varied screens often only partially overlap due to differences in genotoxic mechanism [8]. Thus, there remains a need for new assays that can be used to discover additional genotoxic compounds.

To address this need, our laboratory recently developed the ATAD5-luciferase HTS assay [9], which exploits the stabilization of the ATAD5 protein following DNA damage [10]. This assay is robust and reproducible in a 1536-well plate format and exhibits a high specificity for genotoxic compounds. Most importantly, in a pilot screen of approximately 4,000 small molecules, the ATAD5-luciferase assay successfully identified three potential chemotherapeutic agents that offer improvements over conventional cancer drugs. These compounds, the antioxidants resveratrol, genistein, and baicalein, can kill rapidly dividing cells without inducing mutagenesis or chromosomal alterations, side-effects that may make cells more resilient to cell-cycle checkpoints or apoptosis [9]. Based on the success of this pilot study, we have since used the ATAD5-luciferase assay to screen a collection of 300,000 chemical probes from the Molecular Library Probe Production Centers Network, generating hundreds of hits that may eventually lead to the production of new and superior drugs to fight cancer (unpublished data).

### Screening for Compounds that Target Specific DNA Repair Mutants

Cancer cells often exhibit deficiencies in one of the six major DNA tolerance or repair pathways (base excision repair (BER), nucleotide excision repair (NER), mismatch repair (MMR), homologous recombination (HR), nonhomologous endjoining (NHEJ), and translesion DNA synthesis (TLS)) that protect cells against the accumulation of mutations and genomic instability. For example, 13% of breast cancers [11], 23% of advanced ovarian cancers [12], 6% of cervical cancers [13], and 4% of non–small-cell lung cancers [14] do not express BRCA1, a component of the HR machinery; missense mutations of the *FANCA* gene, whose protein product plays a key role in inter-strand crosslink repair, have been reported in 4-8% of acute myeloid leukemia (AML) patients [15, 16]; and inherited mutations in the DNA MMR genes are thought to be responsible for approximately 5% of the new cases of colorectal cancer diagnosed each year [17]. Given that DNA repair genes could be sequenced during tumor biopsies, therapies that target specific DNA repair mutants may prove to be extremely beneficial in the era of personalized medicine.

In 2009, Takeda and colleagues reported the development of DNA-repair-deficient chicken DT40 cell lines that could be used for high-throughput genotoxicity screening [18, 19], and by extension, for cancer drug discovery. The advantages of the DT40 experimental system are numerous: they have a high efficiency of targeted integration that allows for the generation of genetic mutations with relative ease [20, 21], they display a stable karyotype and short doubling time, they are unable to completely repair any damage induced in the G1 phase of the cell cycle, they have an unusually long S phase, and they grow in suspension [18]. However, despite the fact that the DNA pathways are well-conserved throughout evolution, it remains to be seen whether the results generated from DT40 screens translate to human systems.

An ongoing project in our laboratory involves the use of the DNA-repair-deficient chicken DT40 cell lines in succession with the ATAD5-luciferase HTS described above. We test the genotoxic compounds uncovered by the ATAD5-luciferase assay in a high-throughput manner against a panel of DT40 cells deficient in Polymerase β (BER), Rev3 (TLS), XPA (NER), FANCC (inter-strand crosslink repair), Ku70/Rad54 (HR/NHEJ), and ATM (double-strand break signaling). Because MMR is not represented in this panel due to the diminished viability of DT40 cells lacking this pathway, we also test the ability of the hits from the ATAD5-luciferase assay to selectively kill a human tumor cell line impaired in the expression of the MMR protein MSH2. Additional human DNA repair-deficient cell lines are then used in a secondary screen to validate the compounds that reduced the viability of the DT40 mutants (unpublished data).

### Screening for Compounds that Can Overcome Tumor Chemoresistance

The failure of tumor cells to respond to chemotherapeutic agents presents the largest obstacle in successful cancer treatment. Drug resistance is frequently characterized by a cross-resistance to a number of structurally and functionally distinct agents, even those to which the tumors have never been exposed [22]. The primary mechanism by which tumor cells develop a multidrug resistant (MDR) phenotype involves changes in the expression of transporters that regulate intracellular drug concentrations. Most notable among these transporters is P-glycoprotein (P-gp), a 170 kDa member of the ATP-binding cassette (ABC) superfamily of drug efflux pumps that is known to export several classes of anti-cancer drugs including vinca alkaloids, anthracyclines, taxanes, epipodophyllotoxins, camptothecins, and anthracenes [23-26].

The discovery of ABC transporters and the establishment of MDR hamster, mouse, and human cell lines [27-30] led to enormous efforts beginning in the 1980s to generate inhibitors that could be used to reverse chemoresistance. Unfortunately, many of these first- through fourth-generation compounds performed poorly in clinical trials due to low bioavailability, unexpected secondary physiological effects, and unanticipated drug-drug interactions [31-33]. To address these shortcomings, several groups have designed HTS assays using human MDR cell lines to aid in the discovery of novel inhibitors of P-gp and other ABC transporters. The screening assays range from simple cytotoxicity assays to more complex fluorescence-based assays that utilize labeled substrates [34] or cell lines [35], and have successfully identified several putative reversal agents including mometasone furoate [36], NSC23925 [37], NSC77037 [38], pimozide, acacetin and loxapine [34]. In addition, the ATAD5-luciferase assay revealed two compounds, resveratrol and genistein, that were ultimately shown to selectively kill a P-gp overexpressing KB cell line [9]. Although there is one manuscript that reports an interaction between genistein and the C-terminal nucleotide-binding domain of mouse P-gp [39], no biochemical experiments have ever been conducted to directly test the interaction between resveratrol and P-gp.

Another mechanism that has been implicated in chemoresistance is increased DNA damage tolerance or repair [22], which is often seen following treatment with the genotoxic drugs discussed above [40]. Consequently, several biochemical HTS assays have been carried out to search for inhibitors of the many different DNA repair factors, including PARP-1 [41], Ape1 [42], RecA [43, 44], and Rad51 [45]. In addition, efforts are currently underway in our laboratory to identify inhibitors of the TLS polymerase Polη and the DNA damage response protein ATAD5 using cell-based HTS assays (unpublished data). The assay for Polη inhibitors is based on the observation that polη/polζ deficient chicken DT40 cells, which can tolerate various genotoxic stresses, become sensitive to DNA damaging agents when complemented with human Polη [46]. The screen for ATAD5 inhibitors utilizes the ATAD5-luciferase cell line [9], and is based on the premise that cells with reduced levels of the ATAD5 protein are hypersensitive to DNA damaging agents [10].

## CONCLUSION

Cell-based HTS assays are valuable tools for identifying a variety of potential chemotherapeutic agents, from general and mutant-specific genotoxins to inhibitors of drug efflux pumps and DNA repair factors. Despite the incredible number of new therapeutic options that have been and will continue to be made available through HTS, many impediments to effective cancer treatment still remain: the efficacy of genotoxic agents is limited by their toxicity to normal tissues, the multifactoral nature of chemoresistance negates any benefit obtained by overcoming a single resistance mechanism, and the functional redundancy of the different DNA repair pathways can reduce the effectiveness of repair enzyme inhibitors. Thus, the next challenge will be to determine how the DNA damaging agents, repair inhibitors, and P-gp modulators identified using the assays described above can be used in combination with each other to achieve the best therapeutic outcome.
